# Teachers’ Job Satisfaction and Its Influence on Students’ Academic Performance in Public Secondary Schools: Practical Evidence from Kasese District, Uganda

**DOI:** 10.12688/f1000research.168607.1

**Published:** 2025-09-02

**Authors:** Ashirafu Masudi Kule, Zulaihatu Lawal Bagiwa, Tukur Muhammad, Lucy Aja, Aquila Modupe Otitoju, Kule Jerald

**Affiliations:** 1 Foundations, Kampala International University - Western Campus, Bushenyi, Western Region, Uganda; 2 Foundations, Kampala International University - Western Campus, Bushenyi, Western Region, Uganda; 3 Science Education, Kampala International University - Western Campus, Bushenyi, Western Region, Uganda; 4 Science Education, Kampala International University - Western Campus, Bushenyi, Western Region, Uganda; 5 Humanities, Kampala International University - Western Campus, Bushenyi, Western Region, Uganda; 6 Humanities, Banadir University Somalia, Somalia, Somalia; 7 Humanities, Ibanda University, Ibanda, Uganda

**Keywords:** Teacher Job Satisfaction, Students’ Academic Performance, Public Secondary Schools, Teacher Commitment, and Social Learning

## Abstract

**Background:**

A positive correlation between teacher job satisfaction and student academic achievement is well-established in empirical literature. This link has spurred numerous initiatives to enhance teacher motivation to improve educational outcomes. However, the persistence of poor academic performance in public secondary schools within Kasese District, Uganda, suggests that existing strategies may be insufficient. This disconnect highlights a critical need to investigate the specific dynamics of how teacher job satisfaction influences student performance within this unique rural context.

**Methodology:**

This study employed a mixed-methods design to explore the relationship between teacher job satisfaction and student academic results. Quantitative data were collected through surveys administered to 229 teachers, enabling statistical analysis. To provide deeper contextual insights, qualitative data were gathered through semi-structured interviews with head teachers and focus group discussions with students. This dual approach allowed for both the measurement of the relationship and an understanding of the underlying factors.

**Results:**

The quantitative analysis revealed a statistically significant positive correlation between teacher job satisfaction and student academic performance (r=0.299, p=0.020). Consequently, the null hypothesis, which posited no relationship, was rejected. These findings were supported by qualitative data, which indicated that factors such as teacher motivation, working conditions, and school leadership are pivotal in shaping both teacher satisfaction and, subsequently, student learning outcomes.

**Conclusion:**

The study concludes that teacher job satisfaction is a significant predictor of student academic achievement in Kasese District’s public secondary schools.

**Recommendations:**

It is recommended that a comprehensive approach be adopted to improve educational performance. Key policy reforms should focus on enhancing teacher motivation through fair compensation, transparent career development pathways, and supportive leadership practices. Further research into other context-specific variables is essential for developing effective, targeted interventions in similar settings.

## Introduction

Teacher job satisfaction has long been recognized as a crucial factor influencing teachers’ loyalty to their schools, the quality of instruction, teacher commitment, and student learning outcomes ( Akgün et al., 2023; Tiwari, 2019). It pertains to the emotional state of fulfillment that arises from positive perceptions of working conditions for educators ( Abun, Ubasa, Magallanes, Encarnacion, & Ranay, 2021). Empirical evidence from around the globe indicates that teachers contribute significantly to higher academic performance when they are satisfied with their roles and committed to their schools. Such teachers tend to invest more in lesson planning, adopt innovative pedagogies, and foster supportive classroom environments, all of which enhance student learning ( Aslamiah, 2019; Bello & Oredein, 2022).

In response to these findings, many countries have taken steps to improve teacher satisfaction by reducing administrative burdens and expanding professional development opportunities. For instance, Sweden’s education system has streamlined paperwork to allow teachers to focus more on core instructional tasks, while the USA has integrated academic and career-technical standards to motivate and satisfy teachers, thereby enhancing their performance ( Manley & Manley, 2022; Leal Filho, Viera Trevisan, Dinis, Sivapalan, Wahaj, & Liakh, 2023). In Kenya, the implementation of performance appraisal systems aims to enhance teacher productivity and accountability ( Owuonda et al., 2020). Similarly, Uganda has adopted measures such as salary enhancements and professional development programs to improve teachers’ welfare, notably in Kasese district, to boost teacher satisfaction and encourage positive contributions to student academic achievement ( Namyalo & Sekiswa, 2024; Racheal & Peter, 2023).

In terms of theoretical grounding, the study draws on two key frameworks: the Theory of Organizational Commitment ( Meyer & Allen, 1997) and Bandura’s Social Learning Theory (1977). The former emphasizes that organizational success relies on employees’ psychological attachment to their institutions, which motivates them to demonstrate higher effort and loyalty. It delineates three dimensions of commitment: affective, continuance, and normative, each shaping different motivational drivers that influence teachers’ willingness to invest effort, support instructional activities, and remain committed due to emotional attachment, perceived costs, or a sense of moral obligation. These dimensions are linked to job satisfaction and organizational support, which in turn influence motivation, retention, and performance factors that are critical to improving student achievement through sustained teacher engagement.

Complementing this is Bandura’s Social Learning Theory, which underscores the importance of observational learning, imitation, and modeling in academic contexts. It asserts that students learn behaviors and attitudes by observing teachers, who serve as role models and influence student motivation, discipline, and instructional engagement. Teachers, in turn, adopt instructional practices from school leaders, affecting the quality of teaching and learner outcomes. This bidirectional influence suggests that motivated teachers, supported by organizational commitment, can have a profound impact on student performance through behavioral modeling and reinforcement. The integration of these theories provides a holistic lens to understand how teacher job satisfaction and organizational commitment influence instructional quality and student achievement, addressing a notable gap in previous research that has often studied these issues in isolation or without theoretical integration.

Empirical research consistently underscores the positive association between teacher job satisfaction and student achievement across various contexts. Studies in Nigeria, Pakistan, and Tanzania have shown that satisfied teachers tend to produce better academic results, with satisfaction influenced by factors such as pay, working conditions, and school leadership. For example, teacher pay satisfaction has been linked to higher student exam scores in Nigeria ( Adeyanju et al., 2015), while quality leadership and organizational satisfaction have been associated with improved student outcomes in Korea and private schools in Pakistan ( Chun et al., 2019; Torlak & Kuzey, 2019). Furthermore, intrinsic and extrinsic incentives, including career advancement opportunities and salary increases, are recognized as motivating factors that enhance classroom performance and student gains ( Kachhawa et al., 2018; Wolomasi et al., 2019).

Although substantial evidence from various contexts supports the relationship between teacher factors and student outcomes, notable gaps remain in the existing literature, especially within the Ugandan setting. Most previous studies have concentrated on private schools or limited subject areas, with insufficient focus on public secondary schools. Additionally, many of these studies have relied on descriptive methods or narrow correlational approaches. Importantly, no prior research has integrated both the Social Learning Theory and the Organizational Commitment Theory into a comprehensive framework to explore the link between teacher job satisfaction and student academic performance in Kasese. To fill these gaps, this study adopts a cross-sectional, correlational design to investigate these relationships specifically within the Ugandan public secondary school context. The findings are expected to offer valuable, contextually relevant insights with implications for policymakers and educators, especially in education systems facing similar challenges.

Despite these initiatives, student academic performance in Kasese remains disappointingly low, an issue underscored by examination reports showing only 6.3% of students achieving first-grade scores in the UACE exams, with a substantial 28% failing altogether, and only 2% progressing to university education after high school ( UNEB, 2023; Kibalirwandi, 2023). These statistics highlight the need for urgent intervention, as persistent poor performance could lead to increased dropout rates, reduced university progression, low human capital development, and ultimately stunted regional growth. Although existing literature from Nigeria, Pakistan, Tanzania, and other contexts has indicated a correlation between low teacher job satisfaction and poor student performance, there is a notable gap in empirical research specific to Uganda particularly in the public secondary schools of Kasese district ( Adeyanju et al., 2015; Ajani, 2018; Hassan et al., 2020; Iqbal et al., 2016; Jackline, 2018; Shao, 2021).

This study aims to fill that gap by exploring the relationship between teacher job satisfaction and student academic performance in Kasese’s public secondary schools, testing the null hypothesis that no significant relationship exists between these variables. Its findings seek to offer context-specific evidence that can inform policymakers, school administrators, and other stakeholders, providing a useful benchmark for similar regions beyond Uganda. The research is particularly significant because most previous inquiries have employed descriptive methods or focused exclusively on private schools, with few using correlational analyses or exploring secondary education comprehensively. Moreover, past studies have often concentrated on specific subjects or levels of education, neglecting the overall picture in public secondary schools. The current research employs a cross-sectional, correlational design to examine these dynamics thoroughly within Kasese district, aiming to produce actionable insights for improving teacher job satisfaction and student academic outcomes.

### Research objective

To examine the relationship between teachers’ job satisfaction and students’ academic performance in public secondary schools in Kasese District.

## Methodology

### Research design

The study employed a cross-sectional research design, enabling data collection from the sample’s participants at a single point in time ( Wang & Cheng, 2020). This approach was chosen for its relative speed and cost-efficiency in implementation.

### Research approach

A mixed-methods approach was adopted, integrating both quantitative and qualitative techniques to gain a comprehensive understanding of the research issue. Quantitative data provided objective, measurable, and widely applicable insights, while qualitative data offered detailed, contextual perspectives. The combination of these methods facilitated a richer analysis, enabled validation through triangulation, and supported a more nuanced interpretation of complex concepts. Overall, the use of mixed methods enhanced the credibility, depth, and robustness of the study’s findings.

### Target population and sample size

The target population of 1,162 respondents was drawn from eight public secondary schools in Kasese District, picking two schools from each of the four constituencies of Bukonzo West, Bukonzo East, Busongora South, and Busongora North. The population consisted of 8 head teachers, 246 teachers, and 908 senior four students. Senior four students were selected because they had spent four years in school and could provide credible information regarding performance. Students below 18 years were excluded. Therefore, the study employed a sample of 289 respondents using Cochran’s formula (1977) from the target population of 1,162 participants. See Table 1 for study population and Table 2 for sample size determination.

**Table 1.** Population distribution table.

S/NO	School	Head teacher	Teachers	Students	Total
1	A	1	34	145	180
2	B	1	26	106	133
3	C	1	28	114	143
4	D	1	27	96	124
5	E	1	32	125	158
6	F	1	33	120	154
7	G	1	32	100	133
8	H	1	34	102	137
**Total**		**8**	**246**	**908**	**1162**

Source: Kasese District Education Office, 2025.

**Table 2.** Sample size computation and distribution.

	Computation	Sample size	Sampling strategy
School A			Stratified Proportionate Technique
Head Teacher	1	1	Purposive Sampling Technique
Teacher	$\frac{34}{246}\times 60$	8	Random Sampling Technique
Students	$\frac{154}{908}\times 221$	35	Random Sampling Technique
School B			Stratified Proportionate Technique
Head Teacher	1	1	Purposive Sampling Technique
Teacher	$\frac{26}{246}\times 60$	6	Random Sampling Technique
Students	$\frac{106}{908}\times 221$	26	Random Sampling Technique
School C			Stratified Proportionate Technique
Head Teacher	1	1	Purposive Sampling Technique
Teacher	$\frac{28}{246}\times 60$	7	Random Sampling Technique
Students	$\frac{114}{908}\times 221$	28	Random Sampling Technique
School D			Stratified Proportionate Technique
Head Teacher	1	1	Purposive Sampling Technique
Teacher	$\frac{27}{246}\times 60$	7	Random Sampling Technique
Students	$\frac{96}{908}\times 221$	23	Random Sampling Technique
School E			Stratified Proportionate Technique
Head Teacher	1	1	Purposive Sampling Technique
Teacher	$\frac{32}{246}\times 60$	8	Random Sampling Technique
Students	$\frac{125}{908}\times 221$	31	Random Sampling Technique
School F			Stratified Proportionate Technique
Head Teacher	1	1	Purposive Sampling Technique
Teacher	$\frac{33}{246}\times 60$	8	Random Sampling Technique
Students	$\frac{120}{908}\times 221$	29	Random Sampling Technique
School G			Stratified Proportionate Technique
Head Teacher	1	1	Purposive Sampling Technique
Teacher	$\frac{32}{246}\times 60$	8	Random Sampling Technique
Students	$\frac{100}{908}\times 221$	24	Random Sampling Technique
School H			Stratified Proportionate Technique
Head Teacher	1	1	Purposive Sampling Technique
Teacher	$\frac{34}{246}\times 60$	8	Random Sampling Technique
Students	$\frac{102}{908}\times 221$	25	Random Sampling Technique
Total		289	

### Data collection tools

In this study, data were obtained from both primary and secondary sources. Primary data were collected through interviews with head teachers and closed-ended self-administered questionnaires administered to students and teachers. The questionnaire was based on a five-point Likert scale ranging from 1 = Strongly Disagree, 2 = Disagree, 3 = Moderately Agree, 4 = Agree, to 5 = Strongly Agree, with appropriate alternatives provided in each section. Interviewing was guided by an interview guide. Secondary data, on the other hand, were sourced from journals and various open-access databases where pre-existing information was readily available. Primary data provided the most up-to-date and accurate information, while secondary data provided an opportunity for comparison.

### Validity of quantitative

The questionnaire was reviewed and validated by five experts specializing in Education Management and Administration. These experts assessed the instruments for relevance, response consistency, and clarity regarding the study’s objectives. They also examined the items to ensure they aligned with and effectively addressed the research goals. Any necessary corrections suggested by the experts were incorporated into the final version of the questionnaire, particularly in the instructions for data collection.

### Validity of qualitative data

The researchers conducted interviews until the saturation point was reached, when respondents began to repeat their responses in reaction to prompting questions. Additionally, the transcribed data were taken back to the respondents to verify their accuracy. If the participants confirmed that the transcripts accurately reflected their statements, the data were considered valid. In cases where discrepancies were identified, the data were reviewed, and necessary corrections were made.

### Reliability of quantitative data

A pilot study was conducted to assess the feasibility of the research instruments, identify potential flaws, refine ambiguous items, and ensure alignment with the research objectives. Following this, reliability was evaluated using Cronbach’s Alpha, a widely accepted statistical measure for estimating the internal consistency and reliability of the instrument ( Baker, 2020). An alpha coefficient of 0.84 was obtained, indicating a strong internal consistency, which suggests that the data collected through the instrument are reliable and can be confidently generalized to the target population. This approach to reliability testing enhances the credibility of the findings and demonstrates the robustness of the research instrument used in the study.

### Reliability of qualitative data

After transcribing the data, the researcher identified the main ideas and then employed thematic analysis to systematically code and categorize the data. To ensure the reliability of the coding process, the identified themes and subthemes were independently reviewed and scored by an interrater. An interrater reliability test was conducted to assess the consistency of theme and subtheme generation between raters. A reliability index of 0.70 or higher was considered acceptable, indicating a satisfactory level of agreement and consistency in the coding process, thus supporting the validity of the qualitative analysis for the research objectives.

### Methods of data collection

For the collection of quantitative data, a structured questionnaire was employed to gather information from participants regarding teachers’ attitudes towards job satisfaction and their influence on students’ academic performance in public secondary schools within Kasese district. The use of questionnaires was justified by their efficiency in collecting data from a large sample, enabling the straightforward statistical analysis of responses to identify patterns, trends, and correlations. Additionally, this approach is cost-effective and time-efficient, making it particularly suitable for capturing a broad perspective on the research variables and facilitating a comprehensive understanding of the phenomena under investigation. To obtain qualitative insights, semi-structured interviews with head teachers and focus group discussions with students were conducted. The interviews were designed as one-on-one sessions, allowing participants to express their views, experiences, and perceptions in their own words, with responses recorded and later transcribed for thematic analysis. Focus group discussions provided a platform for moderated, interactive conversations among groups of students, encouraging the exchange of diverse perspectives and collective insights. Both qualitative methods yielded rich, detailed data that were analyzed thematically to explore deeper meanings and contextual factors relevant to the research objectives.

### Methods of data analysis

This study was conducted in eight public secondary schools in Kasese District, with the distribution of head teachers, teachers, and students across schools summarized in Table 1. A stratified sampling strategy allocated the sample across respondent groups and schools, and sample size computation and allocation by stratum are shown in Table 2. Data collection involved questionnaires administered to teachers and students, alongside semi-structured interviews and focus group discussions with head teachers and students; achieved sample sizes and response rates by group are presented in Table 3. Quantitative data were analyzed in IBM SPSS Statistics version 25.0 using both descriptive and inferential techniques: results were produced at a significance level of 0.05, descriptive findings were displayed in frequency distribution tables, and inferential analyses employed the Pearson product–moment correlation coefficient and multiple regression to determine the strength of linear relationships and the extent to which independent variables contributed to variation in the dependent variable. Qualitative data were analyzed thematically: audio recordings were transcribed verbatim; transcripts were coded and categorized according to the study’s objectives and emerging themes; accuracy and validity were enhanced through respondent verification (member checking) with discrepancies reviewed and corrected; and interrater reliability was examined by involving multiple reviewers to confirm consistency in the development of themes and sub-themes aligned with the study’s aims.

**Table 3.** Response rate.

Participants	Sample size	Actual (retrieved)	Response Rate %
Teachers	60	60	100
Headteachers	8	8	100
Students	221	169	76
Total	289	237	82

## Results and analysis

The findings of the study are discussed below:

### Response rate

Initially, the study was planned to collect data from 289 respondents that were 60 teachers for the questionnaire survey, 221 students for the focus group discussion, and 8 head teachers for interviews. However, complete data for both qualitative and quantitative were 237 respondents who included 169 students, 60 teachers, and 8 head teachers, as summarized in Table 3 below. This high response rate suggests that the data collected is reliable and adequately represents the perspectives of teachers, Headteachers, and students regarding teachers’ job satisfaction and students’ academic performance in public secondary schools in Kasese, Uganda. Therefore, the findings derived from these responses provide a strong basis for analyzing the study’s objectives and drawing meaningful conclusions. See Table 3.

### Background characteristics

The background information of teachers in selected public secondary schools in Kasese District provides essential insights into their demographic characteristics and professional experiences, which are fundamental in understanding their perspectives on job satisfaction and academic performance. The data on the background characteristics of respondents are given in Table 4 below. Table 4 indicates that most teachers (41.7%) were aged 30-39, followed by those aged 40-49 (36.7%). Fewer were aged 20-29 (10.0%) and 50+ (11.7%). The majority of the teachers were female (56.7%), with males only 43.3%. Regarding experience, most teachers were 16-20 years (31.7%), while others had 6-10 years (21.7%) or over 20 years (21.7%). The majority (78.3%) taught in grant-aided schools, while 21.7% were in seed schools. The distribution of teaching subjects shows 31.7% were in basic sciences, 21.7% in humanities, 15.0% in languages, and 3.3% in vocational studies.

**Table 4.** Respondents’ background characteristics of teachers.

Categories					Descriptive Mean; χ
Age	20-29 years. N = 6 (10.0%)	30-39 yrs. N = 25 (41.7%)	40-49 years N = 22 (36.7%)	50 ≥ N = 7 (11.7%)	2.5
Gender	Male N = 26 (43.3%)	Female N34 (56.7%)			1.56
Experience	6-10 years N19 (21.7%)	11-15 years. N09 (14.7%)	16-20 years N19 (31.7%)	21 & above N13 (21.7%)	1.56
School Type	Grant-aided N47 (78.3%)	Seed/community N13 (21.7%)			1.2116
Current Teaching Subject	Basic Science N19 (31.7%)	Humanities N13 (21.7%)	Languages N17 (15.0%)	Vocational studies N2 (3.3%)	2.700

The background statistics provide essential context for understanding how teachers’ job satisfaction influences academic performance in public secondary schools in Kasese District. For example, the predominance of teachers aged 30-39 suggests a relatively young and energetic workforce, which may be more adaptable to innovative leadership approaches, while the varying years of experience suggest differing levels of satisfaction and responsiveness to motivation interventions. The dominance of basic science teachers indicates potential leadership focus on STEM-related academic improvements, which may depict the recent intervention to attract more scientists through the enhancement of their salaries to improve their job satisfaction. Lastly, the distribution of background characteristics reflects a diverse representation of teachers across various age groups, experience levels, and subject areas. This diversity ensures a well-rounded collection of perspectives, which enhances the reliability of the findings since it is an incorporation of insights from educators with different professional backgrounds and experiences. These factors shape how leadership influences teacher motivation, satisfaction, instructional quality, and ultimately, student performance. See Table 4.

### Results for descriptive statistics on teachers’ job performance

Descriptive statistics offered valuable context by illustrating teachers’ perceptions regarding their job performance and academic performance, facilitating the subsequent inferential analysis. The assessment of job performance was conducted using 13 different items, each rated on a five-point scale from 1 to 5 (where 1 = Strongly Disagree, 2 = Disagree, 3 = Moderately Agree, 4 = Agree, and 5 = Strongly Agree). The results for these items are summarized in Table 5 below.

**Table 5.** Descriptive statistics of teachers’ views on job satisfaction and students’ academic performance.

	Statement	(1) SD	(2) DA	(3) MA	(4) A	(5) SA	(χ) Mean	St.dv’t
TC01	I am satisfied with my current salary	N = 8 13.3%	N = 15 15.0%	N = 11 18.3%	N = 21 35.0%	N = 11 18.3%	3.300	1.3059
TC02	My salary is adequate to meet my basic needs	N = 1 1.7%	N = 25 41.7%	N = 11 18.3%	N = 7 11.7%	N = 16 26.7%	3.200	1.2876
TC03	My salary is competitive compared to others …	N = 17 28.3%	N = 15 25.0%	N = 5 8.3%	N = 9 15.0%	N = 14 23.3%	2.800	1.5759
TC04	I feel that my salary reflects my contributions to the school	N = 11 18.3%	N = 12 20.0%	N = 10 16.7%	N = 13 21.7%	N = 14 23.3%	3.1107	1.4507
TC05	I believe there is a clear and fair promotion in place	N = 5 8.3%	N = 13 21.7%	N = 11 18.3%	N = 17 28.3%	N = 14 23.3%	3.3667	1.2208
TC06	I feel that promotions are based on merit and performance	N = 1 1.7%	N = 15 25.0%	N = 8 13.3%	N = 17 28.3%	N = 19 31.7%	3.633	1.3523
TC07	I have adequate opportunities for PD to enhance my career	N = 11 18.3%	N = 4 6.7%	N = 13 21.7%	N = 21 35.0%	N = 11 18.3%	3.285	1.0762
TC08	I’m satisfied with the recognition and rewards I receive for my work	N = 2 3.3%	N = 4 6.7%	N = 16 26.7%	N = 18 30.0%	N = 20 33.3%	3.85	1.040
TC09	Non-promotion of teachers positively influences classroom civility and encourages teachers to accept and work towards improving	N = 16 26.7%	N = 6 10.0%	N = 10 16.7%	N = 15 25.%	N = 13 23.5%	3.050	1.5229
TC10	Teachers’ commitment is influenced by their overall job satisfaction and working conditions	N = 4 6.7%	N = 21 35.7%	N = 8 13.3%	N = 14 23.3%	N = 13 21.7%	3.1833	1.3082
TC11	Teachers are satisfied with their overall work environment	N = 4 6.7%	N = 13 21.7%	N = 5 8.3%	N = 16 26.7%	N = 22 36.7%	3.65	1.358
TC12	Teachers feel that their contributions are valued and recognized by the school admin	N = 7 11.7%	N = 17 28.3%	N = 6 10.0%	N = 2 3.3%	N = 28 46.7%	3.4	1.594
TC13	The salary and benefits teachers receive are fair and meet their expectations	N = 5 8.8%	N = 6 10.0%	N = 18 30.0%	N = 10 16.7%	N = 21 35.0%	3.60	1.297
	**Pooled mean**	**N = 7 11.7%**	**N = 12.420.6%**	**N = 9 15.6%**	**N = 15.2 25.3%**	**N = 15.5 25.9%**	**3.57**	**1.489**

Overall, teachers’ job satisfaction hovered around a neutral to slightly positive level, with a pooled mean score of 3.57. Responses ranged from disagreement to strong agreement. For instance, satisfaction with salary (TC01) had an average of 3.30, with 53.3% of teachers agreeing or strongly agreeing that they were satisfied with their pay, although 28.3% expressed dissatisfaction. The perception of salary adequacy for basic needs (TC02) scored a mean of 3.20, with 38.4% agreement contrasted by 43.4% disagreement. The perception of pay competitiveness (TC03) was relatively low, with a mean of 2.80; over half (53.3%) disagreed that their salaries match those of their peers. Belief that pay appropriately reflects individual contribution (TC04) was slightly above neutral, with a mean of 3.11, showing mixed views; 38.3% agreed, while an equal percentage disagreed.

Promotion opportunities and recognition received more favorable ratings: 51.6% agreed that promotions are fair (TC05, mean 3.37), 60.0% felt that advancement is merit-based (TC06, mean 3.63), and 63.3% expressed satisfaction with recognition and rewards (TC08, mean 3.85). Opportunities for professional development (TC07) garnered an average of 3.29, with a 53.3% agreement rate. Working conditions (TC11) and perceived fairness of benefits (TC13) also received majority support, with mean scores of 3.65 and 3.60, respectively. In contrast, statements related to the links between non-promotion and civility (TC09, mean 3.05), as well as overall job commitment (TC10, mean 3.18), showed less consensus among teachers, reflected in larger standard deviations, which indicated diverse experiences across the sample.

Among the 13 items, the mean scores ranged from a low of 2.80 for salary competitiveness (TC03) to a high of 3.85 for recognition and rewards (TC08). The overall average score of 3.57 suggests a moderate level of job satisfaction among teachers. Variability in responses was captured by standard deviations, which ranged from 1.04 (TC08) up to 1.59 (fairness of benefits, TC12). Items related to pay competitiveness, promotion fairness, and non-monetary rewards tended to have more dispersed responses, indicating diverse experiences, while areas such as recognition (TC08) and professional development (TC07, SD = 1.07) showed greater consensus. In summary, teachers generally feel supported in aspects like merit-based promotion and recognition, but many remain divided over the adequacy and competitiveness of their salaries, possibly reflecting discrepancies between salary levels for humanities and science teachers, the latter of whom earn considerably more. These quantitative findings are corroborated by insights from focus group discussions and interviews, where responses largely align with those summarized in Table 5.

### Descriptive statistics for students’ academic performance

The results related to students’ academic performance are summarized in Table 6, which presents data using frequencies, percentages, means, and standard deviations. Across all eleven items, a significant majority of teachers expressed agreement or strong agreement that their job satisfaction positively influences student performance. Agreement levels ranged from 73.8% to 92.7%, with mean scores falling between 4.03 and 4.37. For instance, item SA01 had 82.3% agreement (mean 4.10), SA02 88.2% (4.15), SA03 89.7% (4.35), SA04 92.7% (4.25), SA05 79.4% (4.30), SA06 89.7% (4.30), SA07 80.9% (4.37), SA08 89.7% (4.22), SA09 82.4% (4.32), SA10 80.9% (4.03), and SA11 73.8% (4.25). The standard deviations, consistently below 1.2 and frequently under 1.0, suggest a strong consensus among teachers. Notably, teachers rated their own engagement and professional development impacts highly, with means of 4.35 and 4.36, respectively. The connection between school facilities and student performance also received robust ratings, averaging 4.25. These findings reinforce the perception that in Kasese’s public secondary schools, factors such as motivation, support, leadership, and skills acquired through training, which contribute to teacher job satisfaction, are seen as key drivers in enhancing students’ academic outcomes.

**Table 6.** Descriptive statistics of students’ academic performance.

#	Statement	(1) SD	(2) DA	(3) N	(4) A	(5) SA	(χ) Mean	St.dv’t
SA01	Students perform better academically when teachers are highly committed	N = 6 8.8%	N = 1 1.5%	N = 5 7.4%	N = 26 38.2%	N = 33 44.1%	4.1000	1.1676
SA02	Teachers’ commitment positively influences students’ motivation to learn and excel	N = 6 8.8%	N = 1 1.5%	N = 1 1.5%	N = 30 44.1%	N = 30 44.1%	4.1500	1.1394
SA03	There is a noticeable improvement in students’ academic results when teachers are more engaged and supportive	N = 1 1.5%	N = 1 1.5%	N = 5 7.4%	N = 28 41.2%	N = 33 48.5%	4.3500	0.81978
SA04	The quality of students’ academic performance is linked to the availability of school facilities like computers. lab, and library	N = 2 2.9%	N = 1 1.5%	N = 2 2.9%	N = 35 51.5%	N = 28 41.2%	4.2500	0.87576
SA05	Teachers’ commitment has a significant impact on students’ overall academic achievement	N = 2 2.9%	N = 1 1.5%	N = 5 7.4%	N = 26 38.2%	N = 34 41.2%	4.3000	0.92608
SA06	Positive school leadership contributes to a more effective teaching environment, which can lead to better students’ academic performance	N = 1 1.5%	N = 00 00%	N = 6 8.8%	N = 22 39.7%	N = 34 50%	4.3000	0.92608
SA07	Enhanced teacher commitment due to professional development positively affects students’ academic performance	N = 1 1.5%	N = 1 1.5%	N = 12 17.6%	N = 27 39.7%	N = 28 41.2%	4.3663	0.783041
SA08	Teachers’ efficiency and commitment lead to better students’ academic performance	N = 00 00%	N = 1 1.5%	N = 10 14.7%	N = 27 39.7%	N = 34 50%	4.2163	0.78321
SA09	Professional development for teachers contributes to improved academic outcomes for students	N = 00 00%	N = 1 1.5%	N = 11 16.2%	N = 31 45.6%	N = 25 36.8%	4.3163	0.78312
SA10	Students’ performance shows significant improvement when teachers apply strategies learnt	N = 00 00%	N = 5 7.4%	N = 8 11.8%	N = 32 47.1%	N = 23 33.8%	4.0333	0.98577
SA11	The commitment of teachers fostered through professional development leads to higher levels of students’ achievement	N = 0 00%	N = 5 7.4%	N = 6 8.8%	N = 31 45.6%	N = 26 28.2%	4.2500	0.98082
	**Pooled mean**	N = 1.6 2.7%	N = 1.7 2.8%	N = 4.6 7.7%	N = 24 40%	N = 28.4 47.3%	4.2560	0.846811

### Inferential statistics

The inferential analysis involved an overall evaluation of the model through model summary statistics, significance testing via ANOVA, and an examination of regression coefficients to understand the impact of the predictor variable.

### Model summary

This section discusses the results of the regression analysis, focusing on several key statistics: the correlation coefficient (R), the coefficient of determination (R ^2^), the adjusted R ^2^, and the proportion of variance in students’ academic performance explained by teachers’ job performance. Additionally, it includes the standard error of the estimate, which indicates the average difference between observed data points and the model’s predicted values.

According to Table 7, the analysis identified a modest positive relationship between the independent variable (teacher commitment) and the dependent variable (students’ academic performance), with a correlation coefficient of R = 0.299. The model explained approximately 9% of the variability in student performance, as indicated by the R ^2^ value of 0.090. After adjusting for the model’s complexity, the adjusted R ^2^ was 0.074, reflecting a slight correction. The standard error of the estimate was 0.674, meaning that, on average, the actual student performance scores deviate from the predicted scores by about 0.674 points on the performance scale. A lower standard error would suggest a better fit, with predictions more closely matching actual observations.

**Table 7.** Summary of the regression model for teachers’ job satisfaction and students’ academic performance.

Model	R	R square	Adjusted R square	Std. error of the estimate
1	.299 ^a^	.090	.074	.67372

^a^ Predictors: (Constant), VAR00068.

The statistical output also indicated that the model is significant (p = 0.020), signifying that teacher commitment significantly predicts student performance. The model explains a small portion of the total variance (2.591 out of 28.917), leaving most variability unexplained (residual = 26.326). The F-value of 5.71 suggests the model’s predictive power is better than a model with no predictors, indicating the relationship is meaningful.

Further, the regression coefficients revealed that the predictor variable (VAR00068, representing teacher commitment) makes a significant contribution to predicting student performance. The unstandardized coefficient (B = 0.241, p = 0.020) indicates that a one-unit increase in teacher commitment corresponds to an approximate 0.241 increase in student performance, assuming other variables are constant. The standardized coefficient (β = .299) reflects a moderate positive relationship, and the t-value of 2.389 with a p-value less than 0.05 confirms the significance of this predictor. The intercept (B = 3.449) suggests that when teacher commitment is zero, the expected student performance score is 3.449.

## Summary

In conclusion, there is a positive but relatively weak relationship between teacher commitment and student grades (R = 0.299), with commitment explaining only around 9% of the variance in student performance (R ^2^ = 0.090; adjusted R ^2^ = 0.074). The average prediction error of the model is approximately 0.67 points, indicating that the model’s predictions could be off by about two-thirds of a grade. Despite this, the F-statistic of 5.71 highlights that the relationship is statistically significant. Essentially, engaged and committed teachers do contribute to improved student performance, but a large portion of the variation in academic success is influenced by other factors such as resources, motivation, and family support, which are not captured by this model. Based on these findings, the null hypothesis stating that no relationship exists between teachers’ job satisfaction and student academic performance was rejected, affirming that a significant association is present in Kasese’s public secondary schools.

### Qualitative findings

The qualitative findings were in agreement with the quantitative results. Interviews with Headteachers reveal that respondents generally hold a strong and positive perception of the influence of teachers’ job satisfaction on students’ academic performance. Many acknowledged that when job satisfaction is low, teachers’ commitment and student academic performance tend to decrease, but when teachers are motivated and their satisfaction and commitment levels improve, they increase their effort, and academic performance improves too. For example, Respondent H03 said, “Student performance has been gradually improving … increased teacher commitment …” Another respondent said, “Teachers still go the extra mile to supervise student projects …” adding that this increases efficiency of instruction and motivates students to perform better.

However, some head teachers reported facing numerous challenges that hinder their ability to motivate teachers. Similarly, teachers encounter obstacles that diminish their job satisfaction factors, which collectively contribute to poor performance in certain schools. For example, one respondent said, “We face several challenges, including inadequate incentives and motivation for teachers … and frequent teacher transfers.” (Respondent H07), while another said, “Teachers often face these challenges alone, which can be discouraging…” (Respondent H04).

Besides, findings from Focus Group Discussions show that students’ voices closely mirror the survey’s overwhelmingly positive scores relating to job satisfaction and academic performance. Students generally express that committed and satisfied teachers increase their motivation, boost their confidence, and encourage them to put in more effort, leading to improved academic performance. Conversely, a lack of teacher investment leads to decreased interest and lower grades. This can be illustrated by some of the students’ responses as below:

*Most of my teachers are involved in our learning. They give us notes, clarify doubts, and sometimes even offer extra lessons after school. When they do that, I always score higher on tests.* (Respondent S03)

Another student said:

*When teachers are committed, I feel more motivated to learn. Their encouragement boosts my confidence and makes me put in more effort. But when they don’t seem invested, I lose interest in the subject, and my grades drop.* (Respondent S05)

Another student explained that when teachers experience job satisfaction, they tend to be more committed and work harder, which in turn leads to improved performance. The student said:

*I think my academic performance is closely tied to how committed my teachers are. When teachers put in extra effort, it’s easier for me to perform well. If they don’t engage with us, I struggle more with the subject.* (Respondent S01)

## Discussion

The findings from the ANOVA results in Table 8 indicated a statistically significant relationship (p = .020) between teacher job satisfaction and students’ academic achievement. This outcome aligns with the study by Adeyanju et al. (2015), who found that teacher pay satisfaction significantly improved student examination performance in Nigeria. Similarly, it agrees with the conclusions of Bello and Oredein (2022), who emphasized that satisfied teachers dedicate more effort to instruction, thereby enhancing student outcomes. In the same vein, the findings are supported by Wolomasi et al. (2019), who demonstrated that both intrinsic and extrinsic factors of job satisfaction motivate teachers and translate into improved student performance.

**Table 8.** ANOVA ^b^.

Source	Sum of squares	df	Mean square	F	Sig.
Regression	2.591	1	2.591	5.71	.020
Residual	26.326	58	.454		
Total	28.917	59			

^a^ Predictor: (Constant), VAR00068.

^b^ Dependent variable: VAR00072.

Furthermore, the regression coefficients reported in Table 9 revealed a positive and significant effect of teacher job satisfaction on student performance. This result is consistent with the work of Chun et al. (2019) in Korea and Torlak and Kuzey (2019) in Pakistan, who found that teacher satisfaction and organizational support significantly boosted academic achievement. It also concurs with Aslamiah (2019), who observed that satisfied teachers are more likely to adopt innovative pedagogical strategies that enhance student learning.

**Table 9.** Coefficient.

Predictor	B	SE	β	t	p
Constant	3.449	0.349	—	9.889	.000
VAR00068	0.241	0.101	.299	2.389	.020

Key: *B* = unstandardized coefficient; SE = standard error; *β* = standardized coefficient.

^a^ Dependent Variable: VAR00072.

However, the effect size obtained in this study (R = .299, R ^2^ ≈ 0.09) was modest, explaining only about 9% of the variance in student performance. This finding contrasts with stronger associations reported by Iqbal et al. (2016) in Pakistan and Ajani (2018) in Tanzania, where job satisfaction accounted for a higher proportion of the variance in student achievement. It also differs from the conclusions of Shao (2021), who observed that teacher satisfaction exerts greater influence when it interacts with other variables such as school leadership and professional training.

Additionally, the regression model in this study focused solely on job satisfaction, whereas Meyer and Allen (1997), through the Organizational Commitment Theory, and Bandura (1977), through the Social Learning Theory, suggest that job satisfaction is most effective when integrated with organizational commitment, leadership, and teacher development. This limitation further contrasts with findings by Owuonda et al. (2020) in Kenya and Racheal and Peter (2023) in Uganda, who highlight that performance appraisals, welfare programs, and school leadership collectively have a greater impact on student achievement than job satisfaction alone.

Qualitative findings, Interview and focus group themes reinforced the quantitative results. Head teachers and students emphasized that students’ performance relies on teachers’ motivation, dedication, and morale, with stronger academic results more likely when teachers are motivated and feel valued.

Implications: Teachers’ job satisfaction is relevant to both teacher well-being and students’ academic pursuits. While the observed association is modest, evidence from both strands suggests that when teachers feel valued through equitable compensation, meaningful professional development, and recognition, they are more likely to invest the time, energy, and emotional labor needed to support students effectively. Addressing teacher satisfaction alongside other systemic supports may help improve academic outcomes in Kasese District.

## Conclusion

The study examined the relationship between teachers’ job satisfaction and students’ academic achievement in public secondary schools in Kasese District. The null hypothesis posited no association between these variables. Findings indicated a statistically significant, though modest, positive relationship; therefore, the null hypothesis was rejected. A simple linear regression indicated that higher teacher job satisfaction was associated with better student academic outcomes (R = .299, p = .020). Regression coefficients, standard errors, and significance levels analyses indicate a statistically significant, albeit modest, positive association between teachers’ job satisfaction and students’ academic performance. Interview and focus group themes reinforced the quantitative results. Head teachers and students emphasized that students’ performance relies on teachers’ motivation, dedication, and morale, with stronger academic results more likely when teachers are motivated and feel valued. Teachers’ job satisfaction is relevant to both teachers’ well-being and students’ academic pursuits. While the observed association is modest, evidence from both strands suggests that when teachers feel valued through equitable compensation, meaningful professional development, and recognition, they are more likely to invest the time, energy, and emotional labor needed to support students effectively. Addressing teacher satisfaction in tandem with other systemic supports may help improve academic outcomes in Kasese District.

### Recommendations

This study sounds a serious call for policymakers to not only measure performance but also invest in interventions that improve the conditions of teachers to make good performance more feasible and sustainable. Therefore, currently, interventions such as reforming promotion pathways, investing in equitable professional development, and addressing salary disparities, especially between science and humanities teachers, could greatly energize teachers and enhance learning outcomes in ways that standardized tests and examinations alone cannot capture. All said and moving forward, exploring the longitudinal impacts of motivational reforms or expanding the exploration to other districts for comparative insights could be a focus for future researchers. In addition, future research could isolate the influence of leadership, community involvement, or learner readiness to more fully unpack the ecosystem of performance, as this would be greatly valuable. In conclusion, one clear message from this study is that when teachers thrive, students also rise.

## Declaration of ethics approval

After being fully briefed on the objectives of the study, they understood the importance of protecting their privacy and the impact of motivation on secondary school teachers’ job performance: an empirical analysis of output planning, monitoring, and review in Kasese district, Uganda. The study was accepted by Kampala International University’s (Uganda) Research and Ethics Committee (REC), with approval number KIU-2024-376 and under the standards and directives of the National Research Ethics Guidelines, registration number with the UNCST is SS3529ES.

## Consent to participate

To ensure that participant consent was obtained and confidentiality was maintained, all participating head teachers, deputy head teachers, directors of studies, and teachers signed an informed consent form to provide written consent for the study. The informed consent form was signed by each participant, as we verified.

## Consent to participate after being informed

To guarantee that participant consent was obtained and confidentiality was maintained, all participating head teachers, teachers, and students signed an informed consent form to provide their written consent. We verified that the informed consent form was signed by each participant.

## Authorization for publication

All authors of Teachers’ Job Satisfaction and Its Influence on Students’ Academic Performance in Public Secondary Schools: Practical Evidence from Kasese District, Uganda gave their approval for this manuscript to be published.

## Contributions from the authors

The contributions from the authors include Ashirafu Masudi Kule (Corresponding author, work design, data interpretation, manuscript approval, and agreement), Zulaiha Lawal Bagiwa (data analysis, review, approval, and agreement), Tukur Muhammad (review, approval, and agreement), Lucy Aja (reviewing, data analysis, approval, and agreement), Aquila Modupe Otitoju (reviewing, analysis, approval and agreement) and Kule Jerald (reviewing, data analysis, approval, and agreement).

## Data Availability

The data from the study “Teachers’ commitment and students’ academic performance in public secondary schools in Kasese district, Uganda.” The researchers uploaded the data to the OSF database and made it publicly available at the OSF database repository with the DOI: https://doi.org/10.17605/OSF.IO/DJNV6 ( Kule et al., 2025).
