# Supplementary material for: Teachers’ Job Satisfaction and Its Influence on Students’ Academic Performance in Public Secondary Schools: Practical Evidence from Kasese District, Uganda
Source: F1000Res. 2025 Sep 2;14:849. [Version 1] doi: 10.12688/f1000research.168607.1 (PMC12831048; doi:10.12688/f1000research.168607.1)
Supplement: Peer review history

# Reviewer report: Reviewer response for version 1

**DOI:** 10.5256/f1000research.185797.r442186

**Published:** 2026-01-23

**Authors:** Anietie E Udokang

**Affiliations:** 1 Statistics, Federal Polytechnic, Offa, Kwara State, Offa, Kwara, Nigeria

**Summary of the paper**

 The paper is a survey carried out to determine the effects of teachers’ job satisfaction on students’ academic performance. A sample size of 289 was arrived at using Cochran’s formula and the teachers and students were randomly selected proportional to the sample size. The methods of data collection used were questionnaire and semi-structured interviews. The study used both descriptive and inferential statistical analysis and applied correlation and regression analysis to establish the relationship between teachers' job satisfaction and students’ academic performance. The results of the analysis revealed a positive relationship. Based on the analysis the study established that there is a significant relationship between teachers’ job performance and students’ academic achievement.

**Evaluation:**

 The right methodology was applied in this study that covers determining the sample size, the method of data collection and analysis of data. The research report (article) was logically/sequentially presented with the topic capturing its objective. In this study the problem was identified and evidence provided to assist policy makers and stakeholders in education sector.

 These not withstanding, the areas of weakness were identified such as considering only one factor that influences students' academic performance which led to the weak R squared and others as shown below.  

**Target Population and Sample Size:**

 This section should include how sample sizes of teachers and students were derived from the sample of 289.

 Tables 1 and 2 should come immediately after this section.

**Table 2: Sample size computation and distribution:**

 The computation of sample size for students in school A is 154 instead of 145 in Table 1, but the sample size of 35 is correct.

**Methods of data analysis:**

 Multiple regression is mentioned here while simple regression is used in the analysis.

 Explain in clear terms how teachers’ job satisfaction and students’ academic performance used in the regression analysis were measured. Also, identify the dependent variable and independent variable in the study.

**Model summary:**

 For more clarity the unit of measurement of the variables should be used. For instance "student performance score is 3.449%".

**Citation:**

 The paper requires more current articles of not more than three years from the date of last publication to be cited.

**Recommendation:**

 Subsequent researches should consider more factors that affect students' academic performance to be able to identify in comparison to their contributions to the performance. The paper by my opinion is approved for indexing after the recommended minor corrections and suggestions.

Is the work clearly and accurately presented and does it cite the current literature?

Partly

If applicable, is the statistical analysis and its interpretation appropriate?

Partly

Are all the source data underlying the results available to ensure full reproducibility?

Yes

Is the study design appropriate and is the work technically sound?

Partly

Are the conclusions drawn adequately supported by the results?

Yes

Are sufficient details of methods and analysis provided to allow replication by others?

Yes

Reviewer Expertise:

Statistics, Data Analytics, Educational Research

I confirm that I have read this submission and believe that I have an appropriate level of expertise to confirm that it is of an acceptable scientific standard.

# Reviewer report: Reviewer response for version 1

**DOI:** 10.5256/f1000research.185797.r440387

**Published:** 2025-12-29

**Authors:** Haoran Ma

**Affiliations:** 1 Fujian Normal University, Fuzhou, China

This paper needs revising two major problems.

 1. The authors need to clearly articulate the connection between this study and previous research, especially given the abundance of studies on satisfaction and academic achievement. My opinion is that authors should not reinvent the wheel unless we have novel insights and knowledge.

 2. Literature review section shows weak. I suggest authors to read the current papers to make improvement. Not only the papers, but also the structure of this section. What's the definition of the core variables showing in this manuscript? And what are the dimensions of them? Did the authors use the scale that is with a good reliability? Experts may play a vital role in it, but the more important thing is the data verification. So please give a result of validity and reliability with full information. Overall it's quite hard to believe the result meets the scientific standard.

Is the work clearly and accurately presented and does it cite the current literature?

Partly

If applicable, is the statistical analysis and its interpretation appropriate?

No

Are all the source data underlying the results available to ensure full reproducibility?

Partly

Is the study design appropriate and is the work technically sound?

Yes

Are the conclusions drawn adequately supported by the results?

Partly

Are sufficient details of methods and analysis provided to allow replication by others?

Partly

Reviewer Expertise:

Teaching and teacher education.

I confirm that I have read this submission and believe that I have an appropriate level of expertise to confirm that it is of an acceptable scientific standard, however I have significant reservations, as outlined above.

# Reviewer report: Reviewer response for version 1

**DOI:** 10.5256/f1000research.185797.r440386

**Published:** 2025-12-23

**Authors:** Ayhan Kandemir

**Affiliations:** 1 Ministry of Education, Bolu, Turkey

The article has a well-chosen topic. However;

 1- The relevant literature, especially the current literature, has not been sufficiently reviewed. The introduction and especially the discussion section should be enriched by reviewing the relevant literature.

 2- Some paragraphs include explanations but no references. References must be added to these sections.

 3- There are some gaps in information and references in the methods section. These must be corrected.

 4- How the qualitative questions were prepared should be made clearer.

 5- The references section should be enriched by taking into account studies conducted in recent years.

 6- If possible, the number of people in the sample should be increased.

Is the work clearly and accurately presented and does it cite the current literature?

No

If applicable, is the statistical analysis and its interpretation appropriate?

Yes

Are all the source data underlying the results available to ensure full reproducibility?

Yes

Is the study design appropriate and is the work technically sound?

Partly

Are the conclusions drawn adequately supported by the results?

Yes

Are sufficient details of methods and analysis provided to allow replication by others?

Yes

Reviewer Expertise:

NA

I confirm that I have read this submission and believe that I have an appropriate level of expertise to confirm that it is of an acceptable scientific standard, however I have significant reservations, as outlined above.

# Reviewer report: Reviewer response for version 1

**DOI:** 10.5256/f1000research.185797.r428439

**Published:** 2025-11-12

**Authors:** Samra Maqbool

**Affiliations:** 1 Beijing Normal University, Beijing, Beijing, China

The study presents a solid investigation into the relationship between teacher job satisfaction and student academic performance in Kasese District, Uganda, using a mixed-methods approach. While the overall design is sound, there are areas requiring further revision:

1. While the sample size is justified, the distribution and methodology for selecting participants from specific constituencies need to be more clearly explained. It would help to detail how the teachers, students, and head teachers from each school were randomly selected to ensure representativeness.
2. The use of regression analysis and ANOVA is appropriate, but the study should better interpret the practical implications of the modest effect size (R = 0.299, R² = 0.090). While statistically significant, the relationship is weak, and the manuscript should address how these findings could influence educational interventions in a practical sense.
3. The qualitative findings are valuable, but they should be integrated more effectively with the quantitative data to provide a more holistic view. The current separation between the two methods can create disconnect in the narrative.
4. The study context (Kasese District) is well-delineated, but more local literature and deeper exploration into regional factors such as resource allocation, local policies, and teacher professional development are necessary to strengthen the analysis.
5. The conclusion needs to better emphasize the multifaceted nature of teacher satisfaction and academic performance. Additional recommendations for targeted interventions based on the findings would be helpful, particularly regarding teacher welfare and leadership practices.

Is the work clearly and accurately presented and does it cite the current literature?

Yes

If applicable, is the statistical analysis and its interpretation appropriate?

Yes

Are all the source data underlying the results available to ensure full reproducibility?

Yes

Is the study design appropriate and is the work technically sound?

Yes

Are the conclusions drawn adequately supported by the results?

Yes

Are sufficient details of methods and analysis provided to allow replication by others?

Yes

Reviewer Expertise:

Education

I confirm that I have read this submission and believe that I have an appropriate level of expertise to state that I do not consider it to be of an acceptable scientific standard, for reasons outlined above.
